# Supplementary material for: Domestication shaped the chromatin landscape of grain amaranth
Source: Nat Commun. 2025 Nov 21;16:10407. doi: 10.1038/s41467-025-66445-w (PMC12644504; doi:10.1038/s41467-025-66445-w)
Supplement: Supplementary file 1 — Supplementary Information [file 41467_2025_66445_MOESM1_ESM.pdf]

Domestication shaped the chromatin landscape of grain amaranth

Graf *et al.*

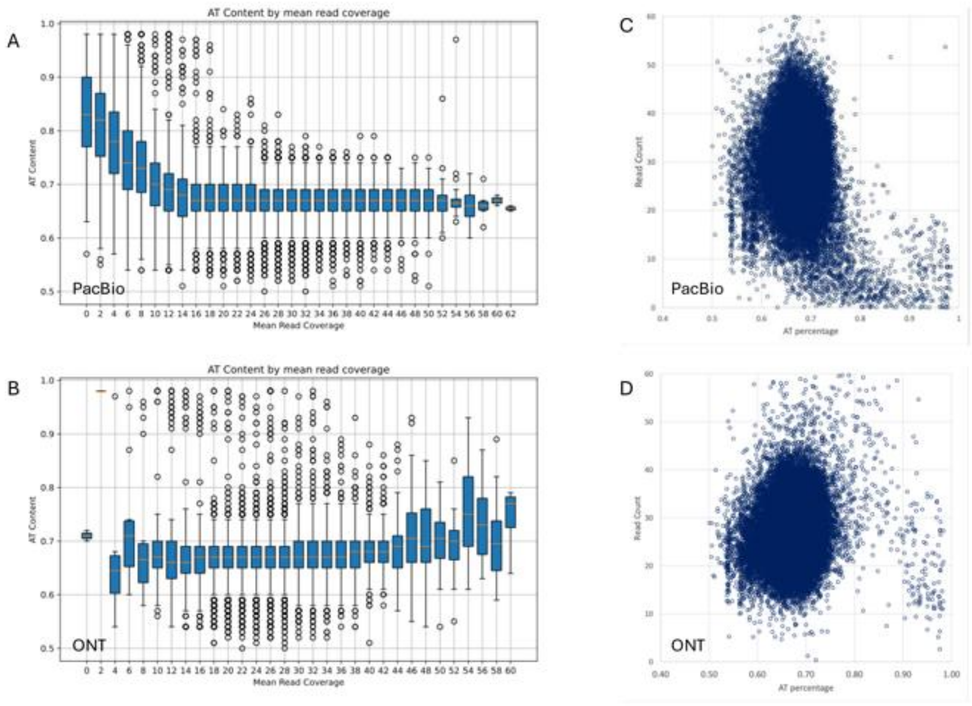

Supplementary Figure 1. Read coverage relative to AT percentage with PacBio HiFi and ONT read types. Panels A (PacBio HiFi) and B (ONT) show the mean read depth (x-axis) at different AT percentage (y-axis) across 10 kb windows of the genome showing the AT bias of the PacBio reads, which leads to more fragmented primary assemblies. Similarly, panels C (PacBio HiFi) and D (ONT) show read count (y-axis) across 10 kb genomic windows with varying AT percentages (x-axis). Note the reduction of PacBio HiFi reads (panel C) in windows with high AT percentage. Amaranths are potentially unique in that they have regions of high AT concentration – at these regions, PacBio HiFi reads often terminate prematurely which then fragments the primary contig assembly. Using a hybrid approach (both PacBio and ONT) successfully resolves this issue.

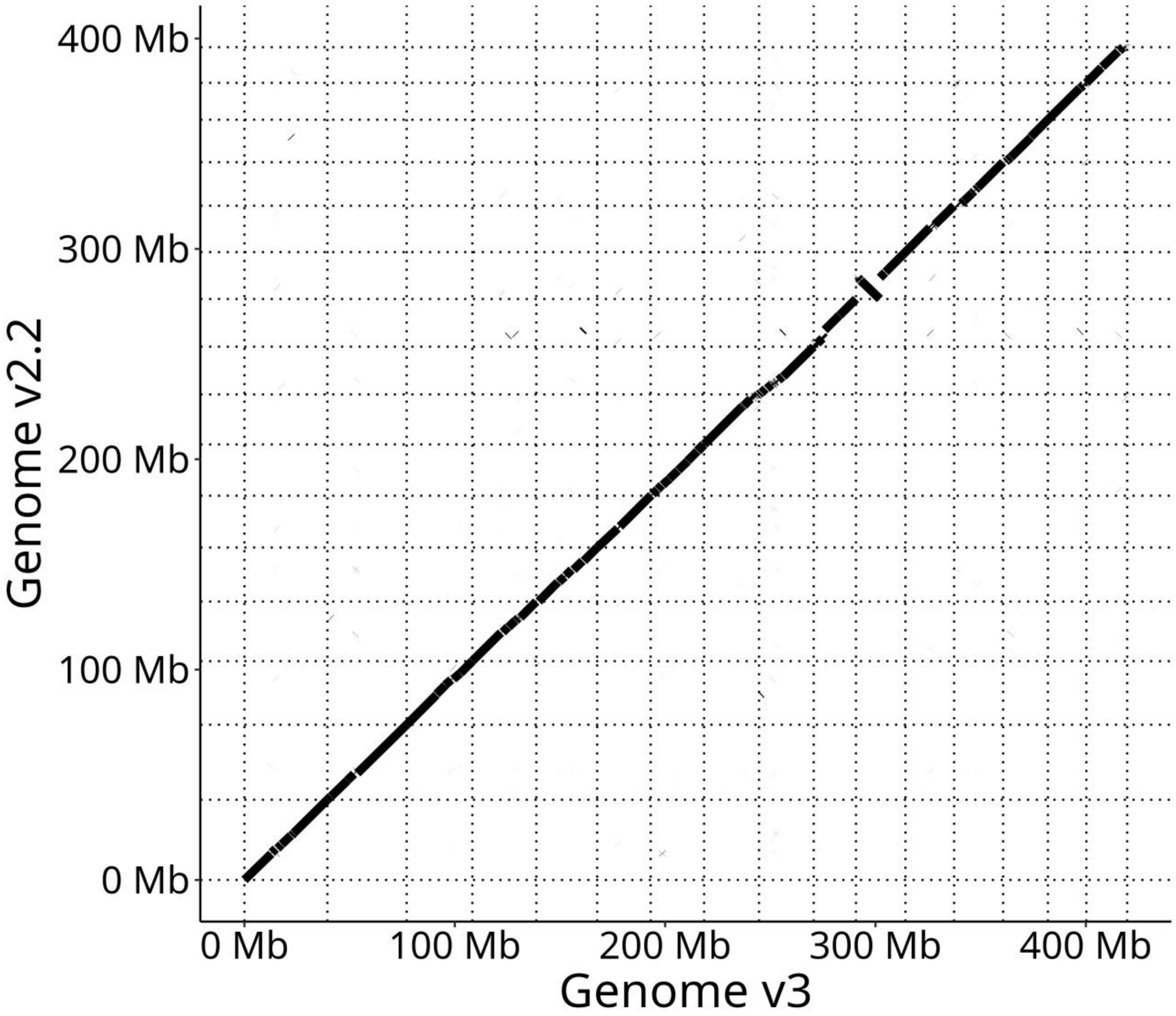

Supplementary Figure 2. Dotplot of whole-genome alignment between *A. hypochondriacus* reference genome versions v2.2 and v3. Only scaffolds corresponding to the 16 chromosomes of *A. hypochondriacus* were depicted.

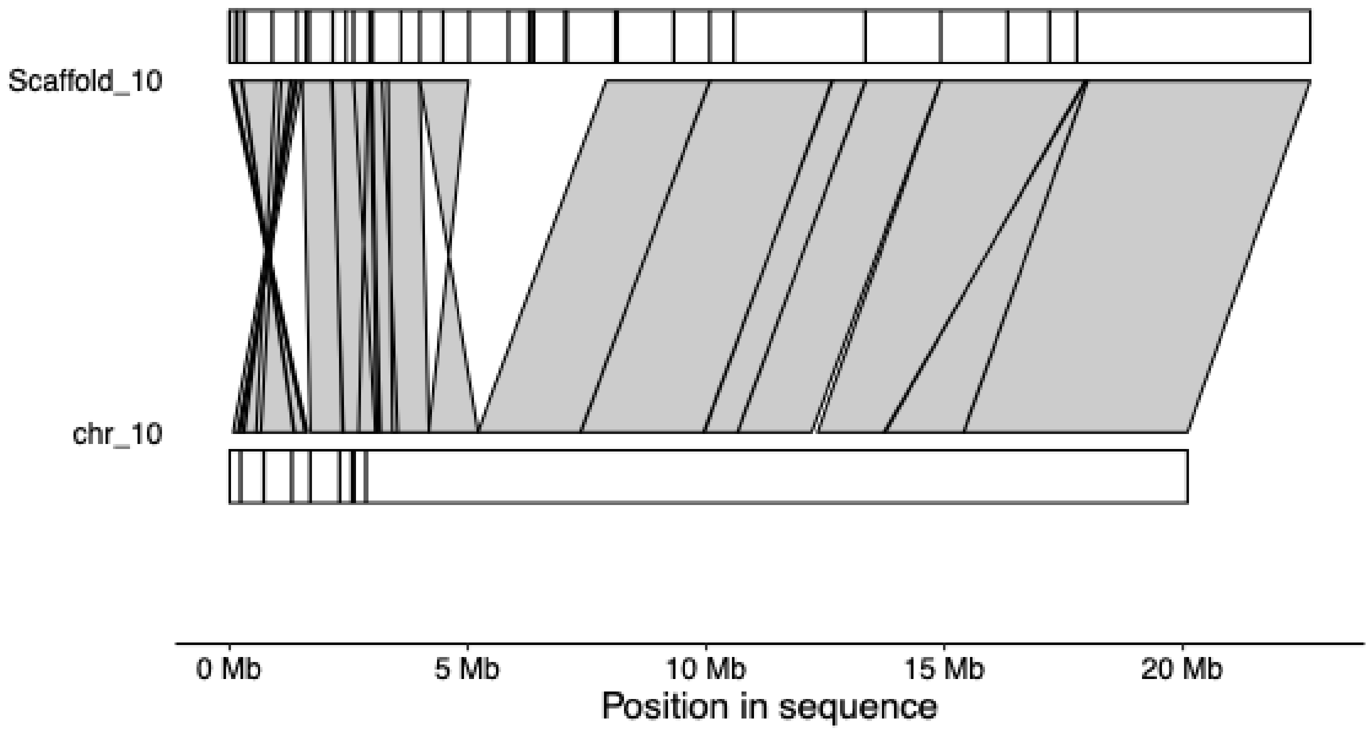

Supplementary Figure 3. Synteny between *A. hypochondriacus* chromosome 10 scaffolds of reference genome versions v2.2 (top) and v3 (bottom). Alignment of scaffold 10 of reference assembly v2.2 and chromosome 10 of reference assembly v3 shows potentially misassembled region missing from the new chromosome assembly. Contig borders of assembly v2.2 and v3 are indicated as vertical black bars on their respective chromosomes.

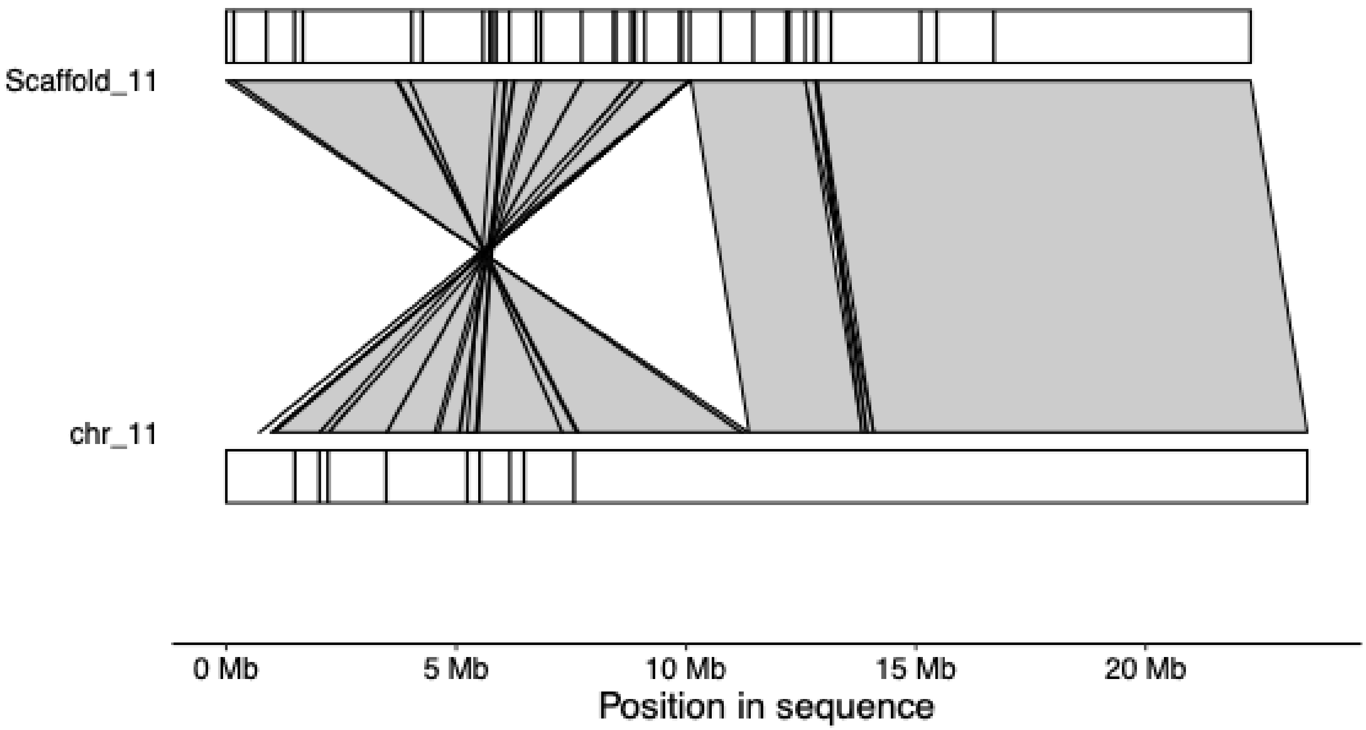

Supplementary Figure 4. Synteny between *A. hypochondriacus* chromosome 11 scaffolds of reference genome versions v2.2 (top) and v3 (bottom). Alignment of scaffold 11 of reference assembly v2.2 and chromosome 11 of reference assembly v3 shows potentially misassembled region inverted between the two assemblies. Contig borders of assembly v2.2 and v3 are indicated as vertical black bars on their respective chromosomes.

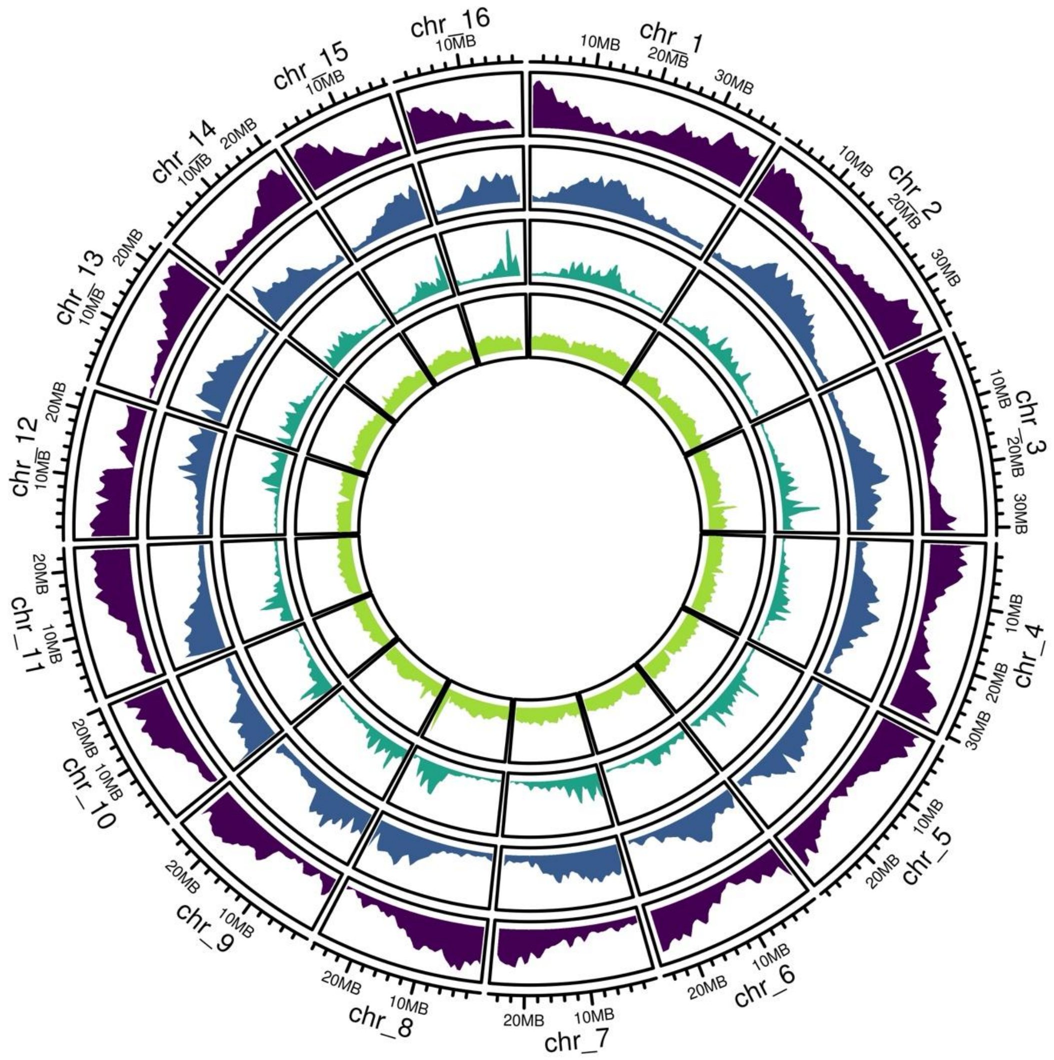

Supplementary Figure 5. Genomic distribution of annotated genes and transposable element classes in *A. hypochondriacus* reference genome v3. Tracks depict (from outside to inside) density of genes, LTR elements, LINEs, and MITEs calculated in 1 Mb windows along the genome.

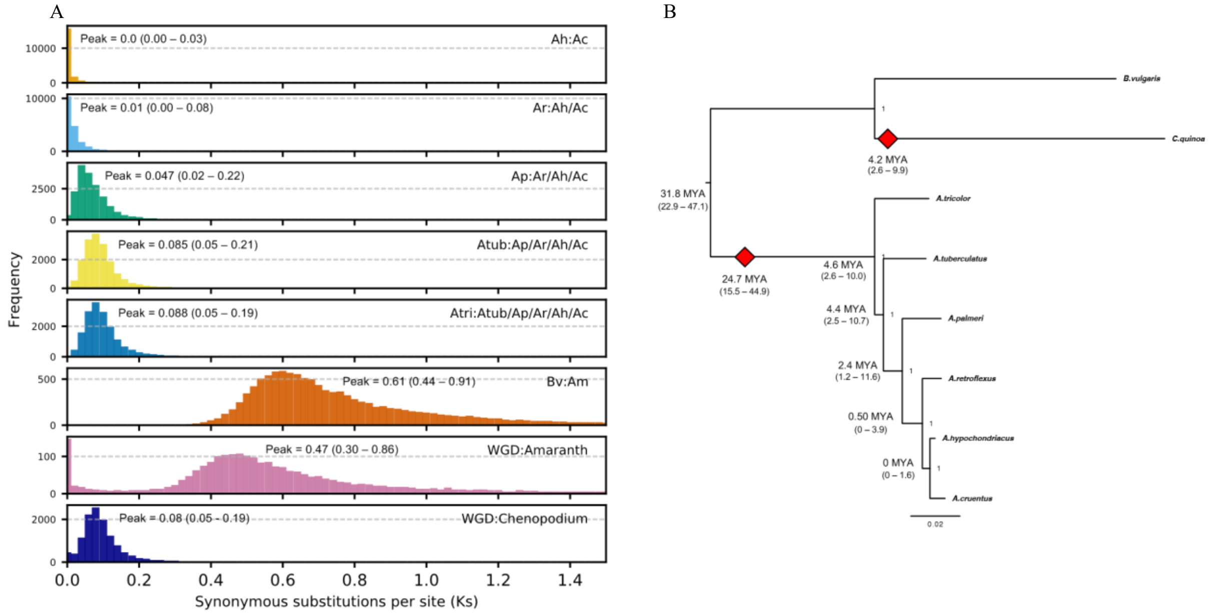

Supplementary Figure 6. Synonymous substitutions per synonymous sites (K_s) distribution identifying whole-genome duplication and speciation and events. A Synonymous substitutions per sites (Ks) histograms. Ks values of syntenic orthologs (speciation events) and paralogs (whole genome duplications) were determined using wgd2[1]. Species abbreviations are *A. tricolor* (Atri), *A. tuberculatus* (Atub), *A. palmeri* (Ap), *A. retroflexus* (Ar), *A. cruentus* (Ac), *A. hypochondriacus* (Ah). Whole genome duplications (WGD) were determined by averaging across all *Amaranthus* species, while the WGD for *Chenopodium* was based solely on *Chenopodium quinoa*. Confidence intervals (95%) are provided in parenthesis. **B** Rooted *Amaranthus* species tree based on conserved orthologs as identified by Orthofinder2[2] showing speciation and whole genome duplications dates as inferred by Ks distributions (see panel A). *B. vulgaris* (Bv) and *C. quinoa* were included as outgroups. Red diamonds indicate estimated points of WGD inferred from peaks in Ks.

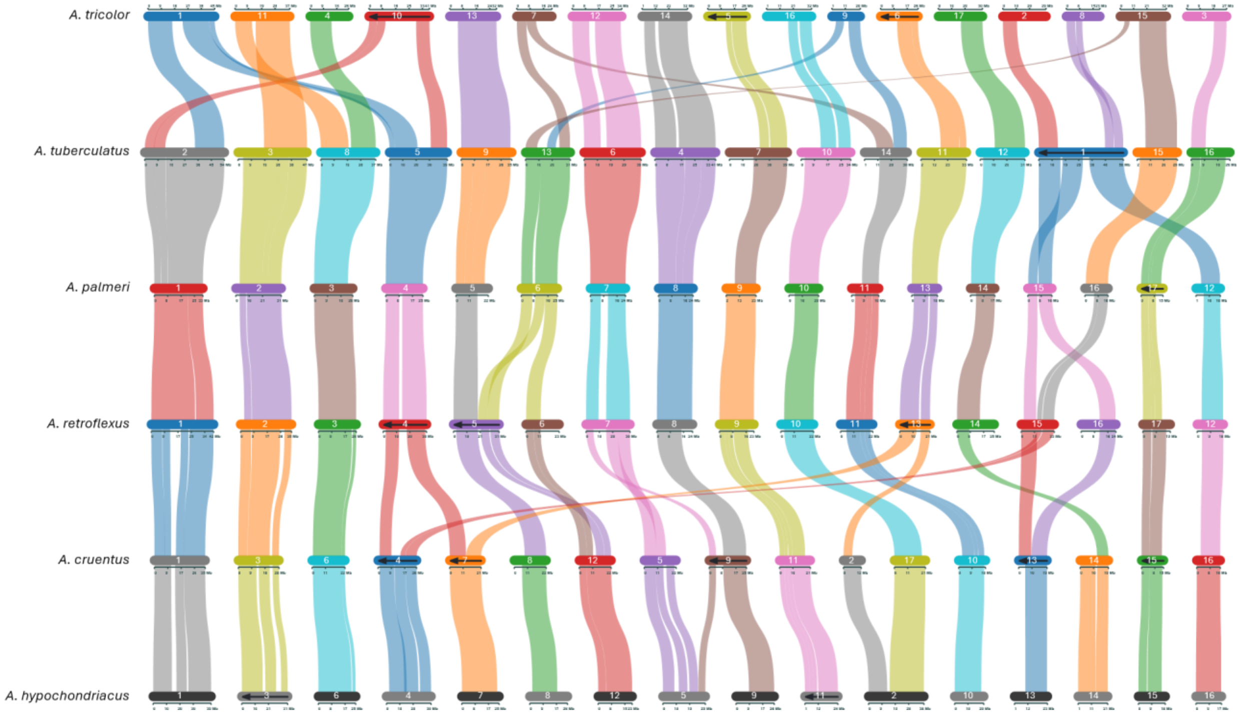

Supplementary Figure 7. Syntenic relationships of orthologous regions among sequenced *Amaranthus* species
Species are ordered vertically by phylogenetic positions (*A. tricolor*, *A. tuberculatus*, *A. palmeri*, *A. retroflexus*, *A. cruentus* and *A. hypochondriacus*). Chromosomes are ordered horizontally, and ribbons are color-coded to show syntenic relationships among chromosomes. Arrows within chromosomes indicate reverse complementation.

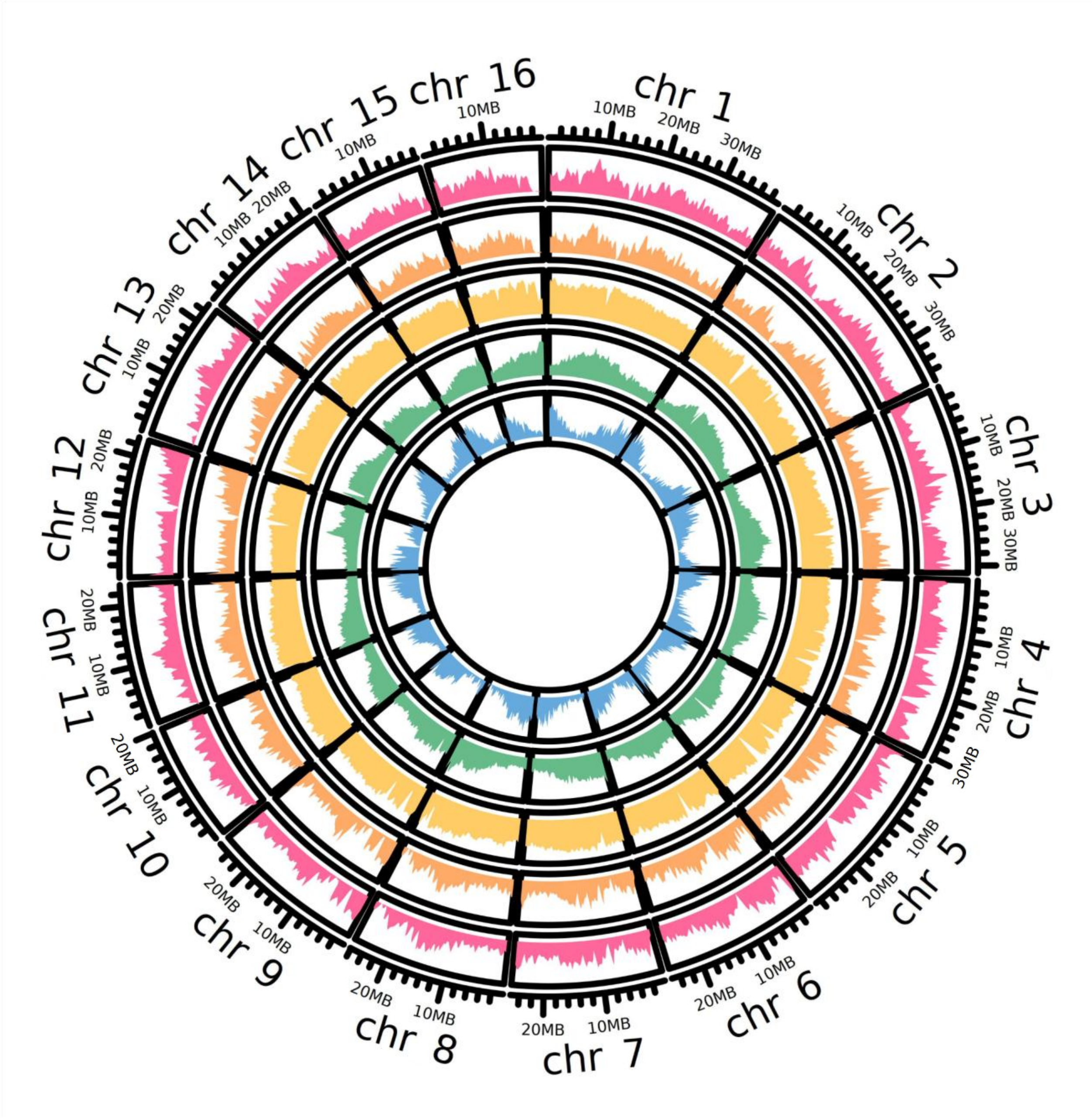

Supplementary Figure 8. Genomic distribution of genes, methylation marks and accessible chromatin regions (ACRs) in *A. hypochondriacus* reference genome. Tracks depict the genomic distribution of (from inside to outside) annotated genes (blue), transposable elements (green), methylation in CpG context (yellow), ACRs found in leaf tissue (orange), ACRs found in seedling tissue (pink). The outer most ring represents the chromosomes of the reference genome and indicates the position within the chromosomes in megabase pairs (Mb).

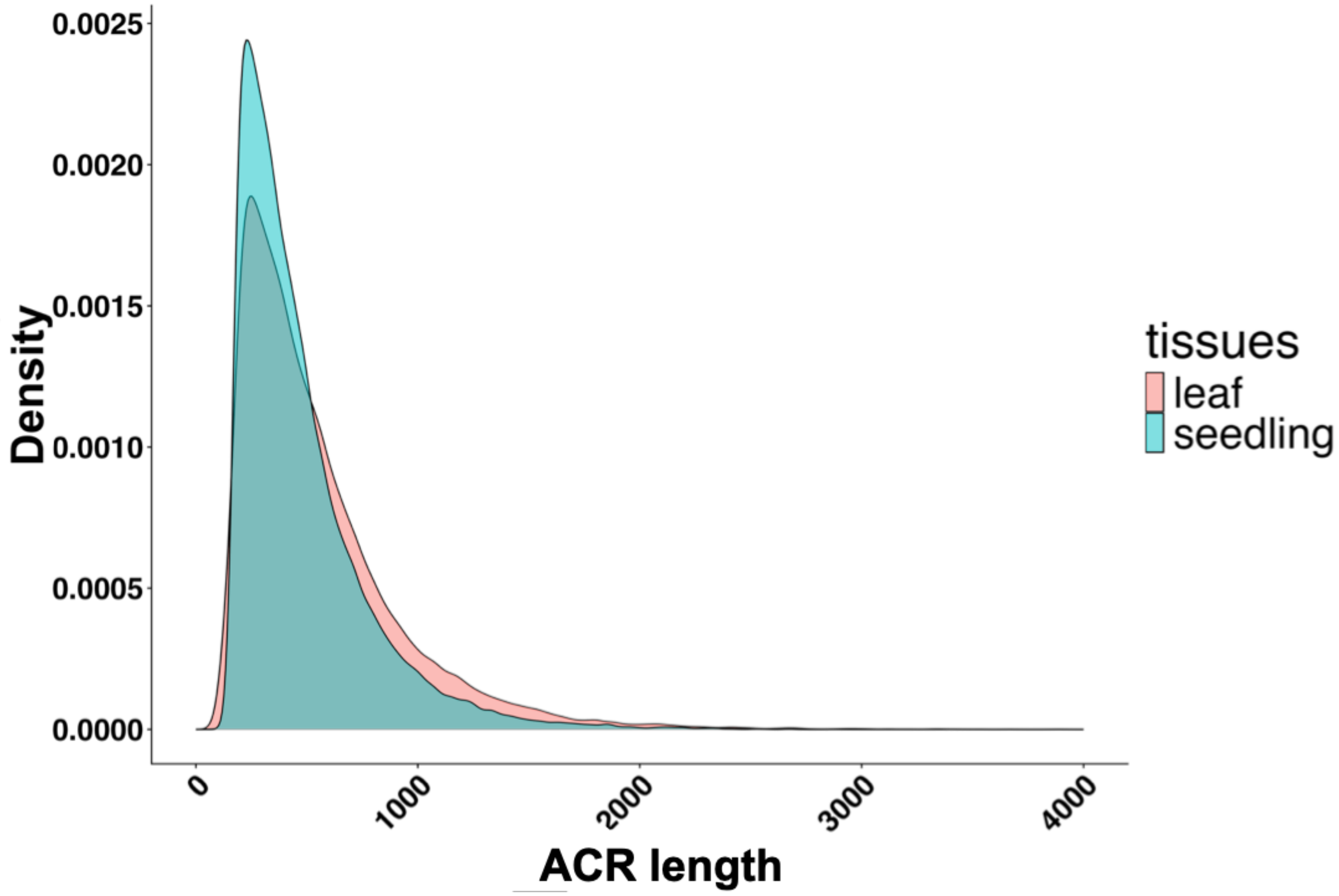

Supplementary Figure 9. Distribution of ACR length in the ATACome. Length of both leaf tissues (pink) and seedling tissue (turquoise) ACRs from samples of PI 558499 aligned to the *A. hypochondriacus* reference genome.

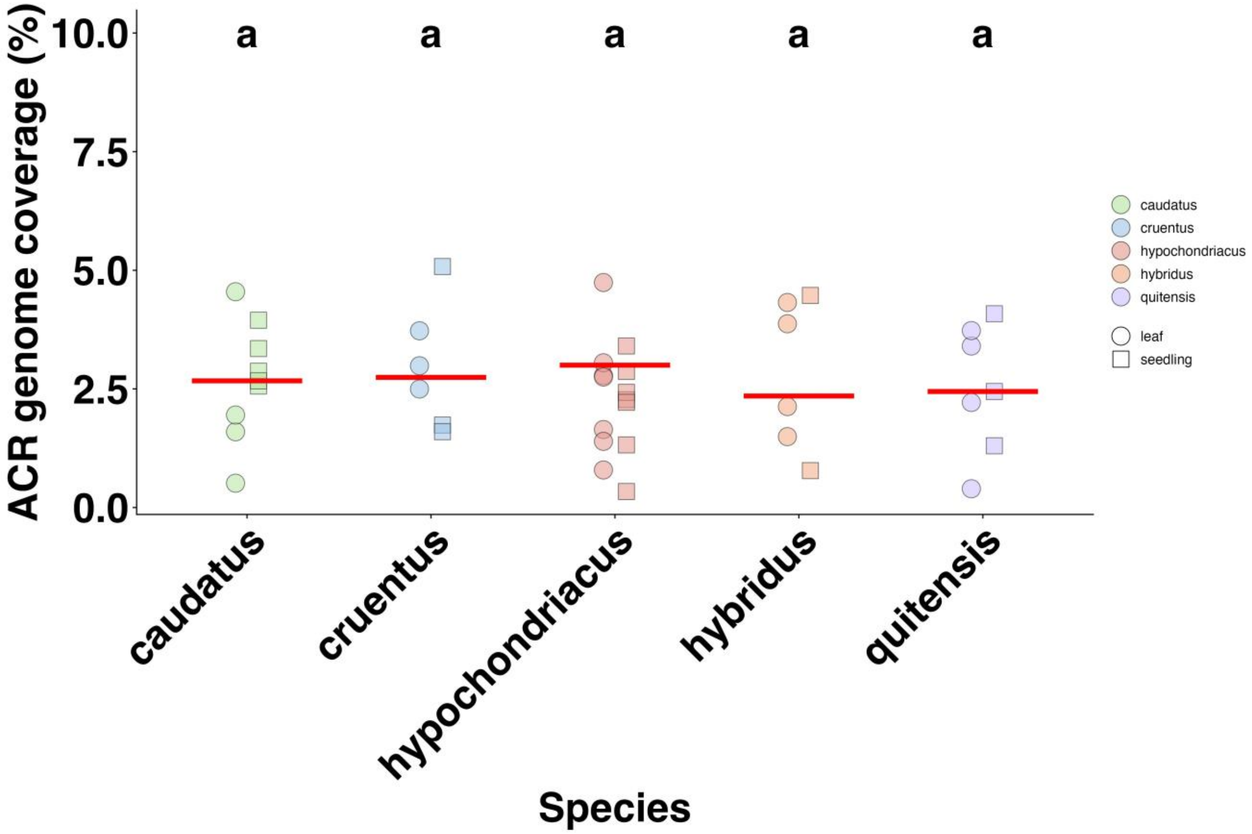

Supplementary Figure 10. Fraction of the *A. hypochondriacus* reference genome covered by the ACRs called in each sample. The tissue and species from which each sample originates is indicated by shape and color, respectively. The mean is indicated by a red line for each species, respectively (*A. caudatus* n=9, *A. cruentus* n=6, *A. hypochondriacus* n= 14, *A. hybridus* n=6, *A. quitensis* n=7). Letters above the species indicate significant differences between groups based on a one-way ANOVA (species=0.821) followed by Tukey's test.

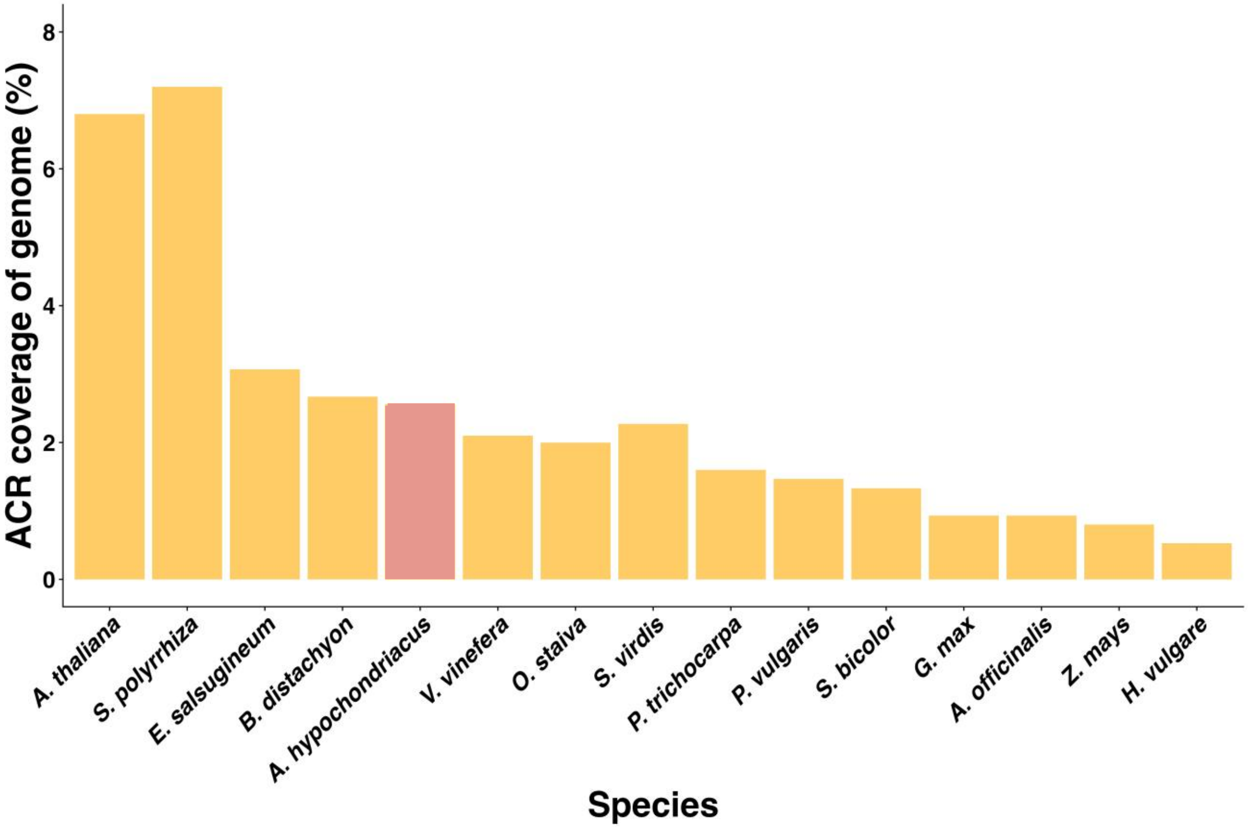

Supplementary Figure 11. Fraction of accessible chromatin relative to genome size in 15 plant species. Y-axis indicates fraction of the whole genome (in %) that was found to be accessible. *A. hypochondriacus* (red) data based on mean coverage of PI 558499 samples, while the other species were plotted based on data from Lu *et al*. [3] and Schwope *et al.* [4].

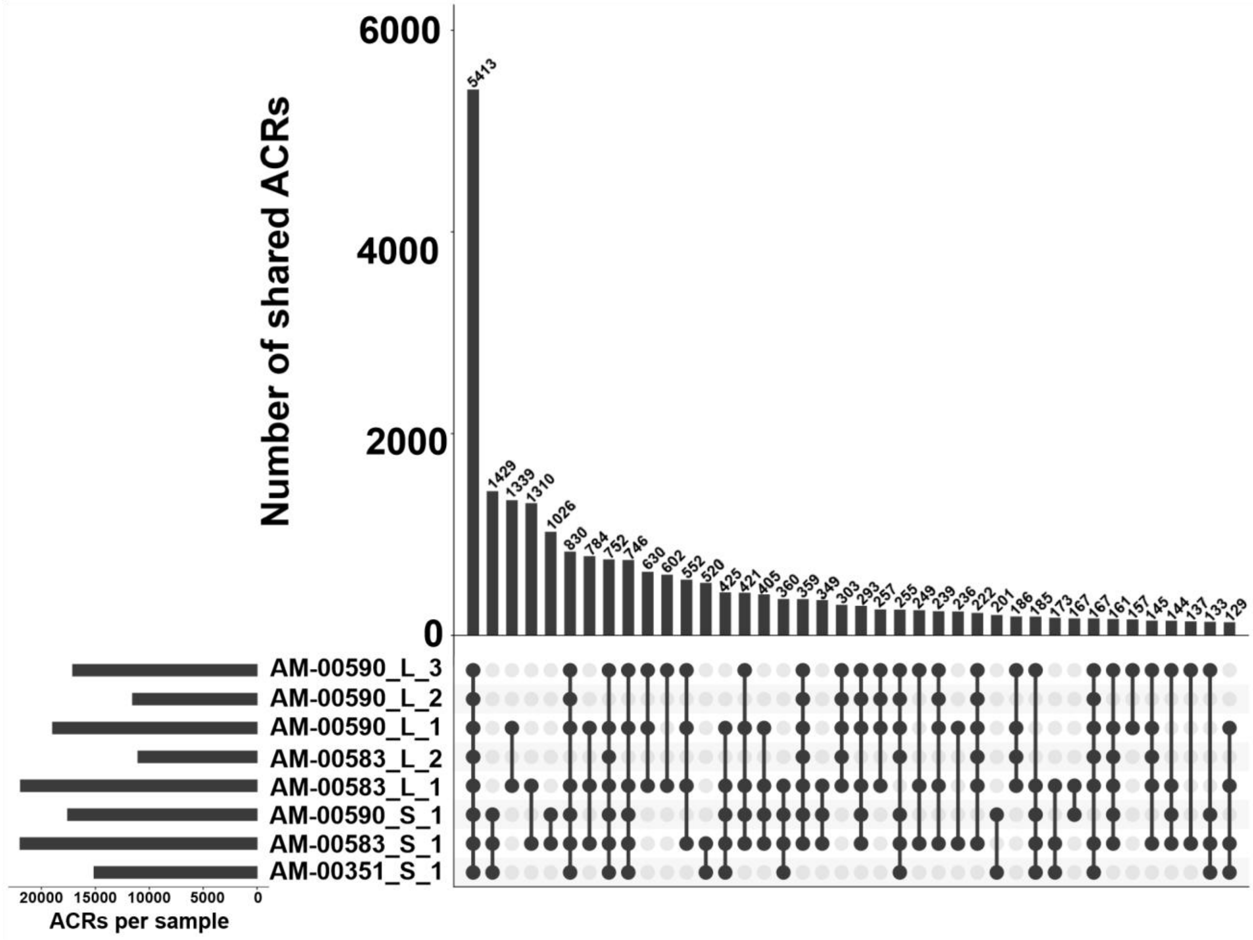

Supplementary Figure 12. Overlap of ACRs between the samples of *A. hypochondriacus*. UpsetR plot of ACRs called in each of the eight samples from PI 558499. The Y-axis of the bar graph indicates the number of ACRs that were shared by samples indicated by joint dots in the corresponding column of the matrix below. ACRs are only part of one column. The second bar graph to the left of the sample names indicates total number of ACRs called in each sample.

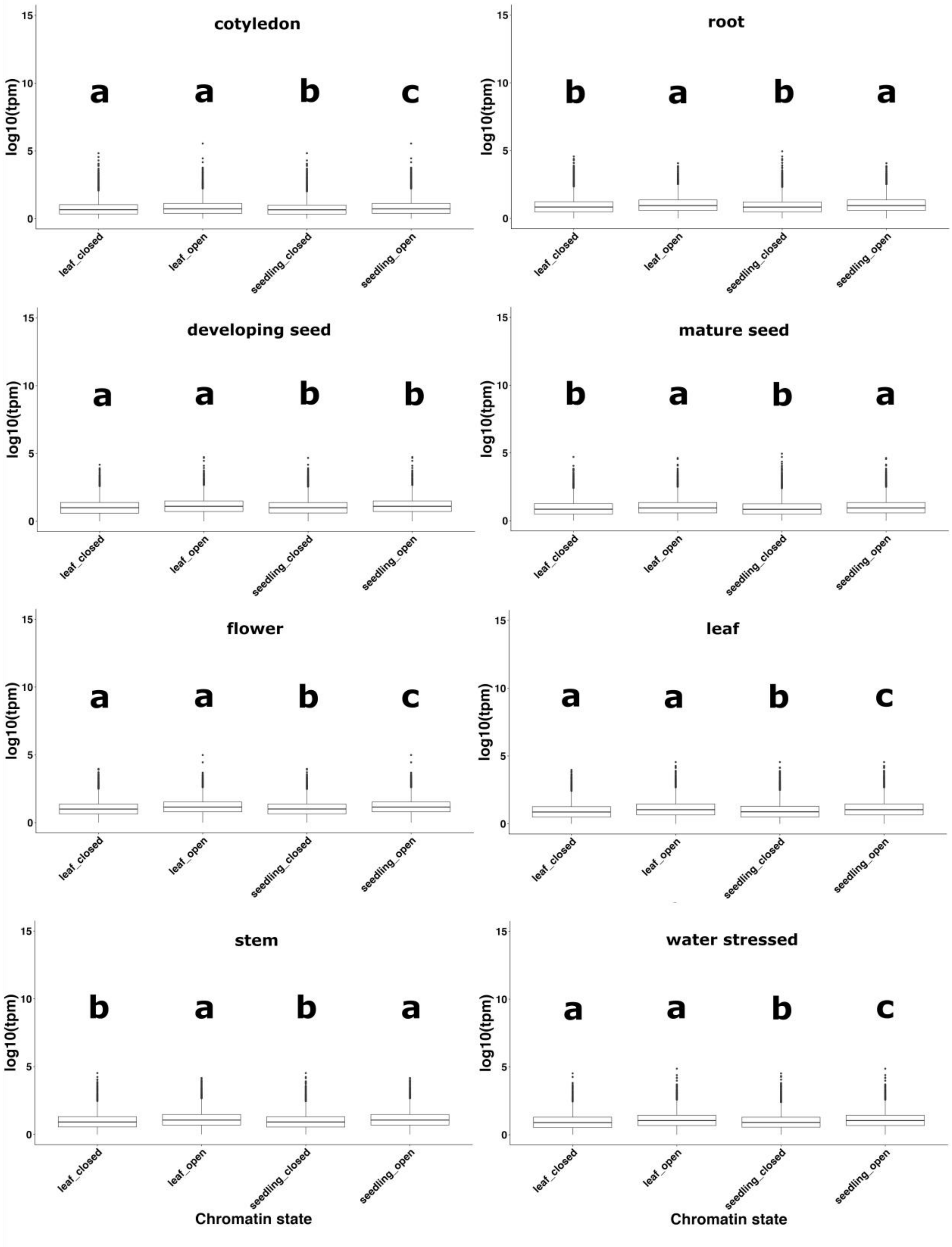

Supplementary Figure 13. Expression comparison in eight tissues. Comparison of expression of genes associated with accessible and closed chromatin in leaf and seedling samples. Genes including promoters (2 kb upstream) that overlapped with at least one ACR were defined as open. An equally sized random subsample of 'closed genes' was taken as comparison. Mean expression values for the genes in each of the eight tissues was taken from Clouse *et al*.[5]. Letters on top of the boxplots indicate significant difference within each of the eight tissues.

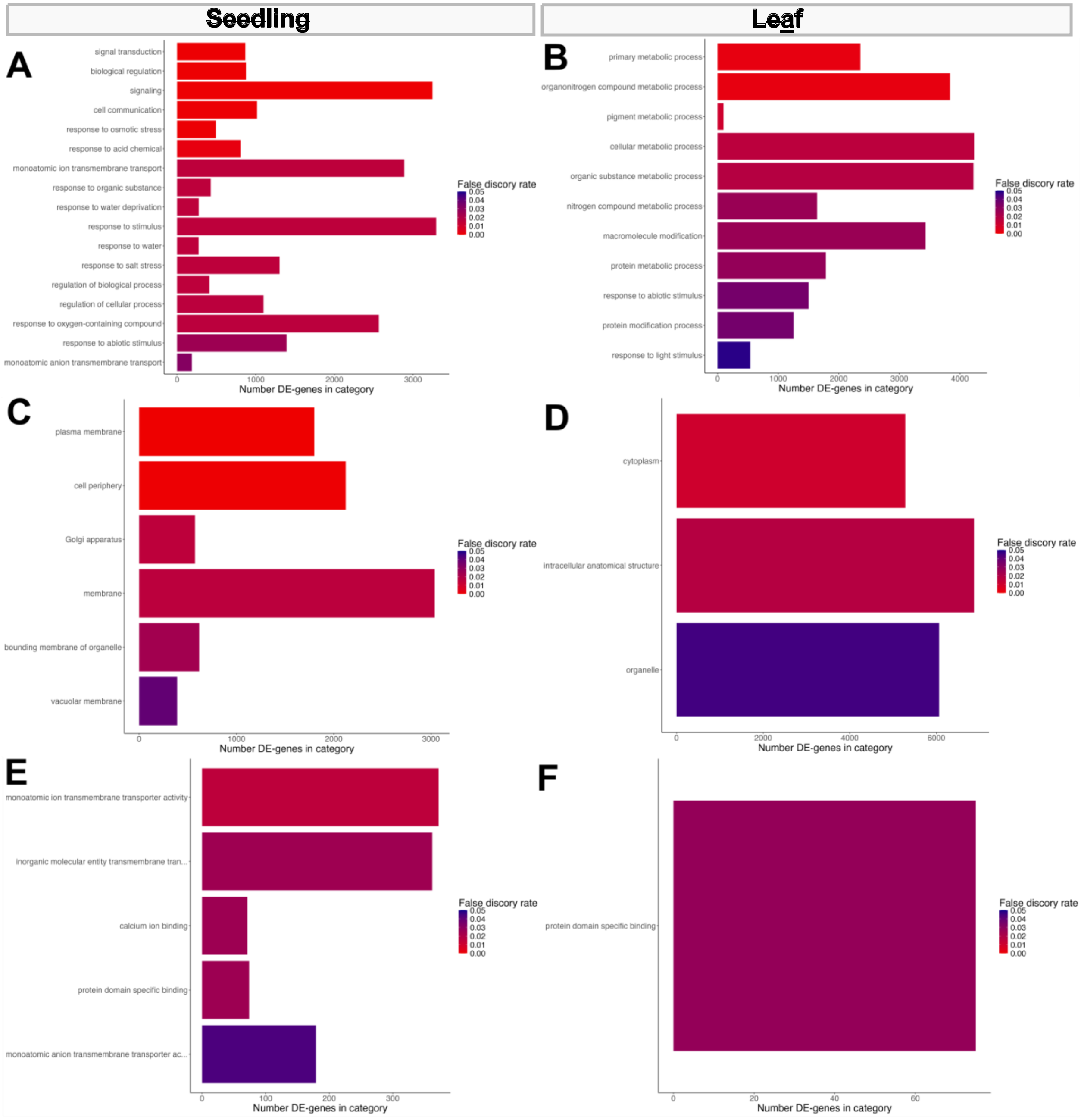

Supplementary Figure 14. Functional categories enriched among genes associated with ACRs in *A. hypochondriacus*. GO-terms enriched among genes associated with ACRs for **A.** biological processes in seedling tissue **B.** biological processes in leaf tissue **C.** cellular components in seedling tissue **D.** cellular components in leaf tissue and **E.** molecular functions in seedling tissue **F.** molecular functions in leaf tissue. False discovery rate cutoff was set 0.05.

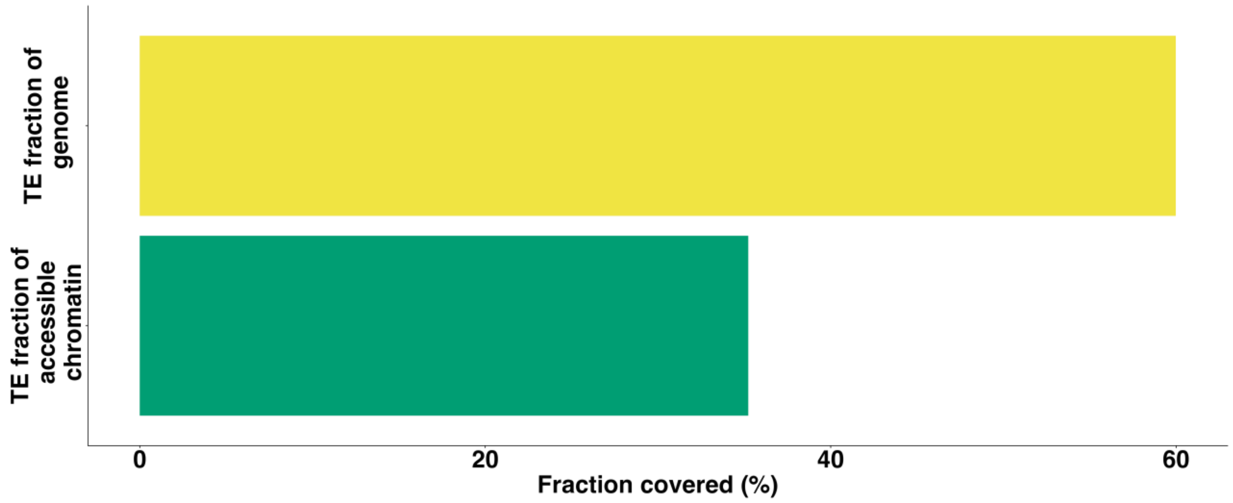

Supplementary Figure 15. TE coverage of open chromatin and the genome. X-axis indicates the fraction of the whole genome (yellow) of *A. hypochondriacus* and accessible chromatin (green) that is occupied by TEs.

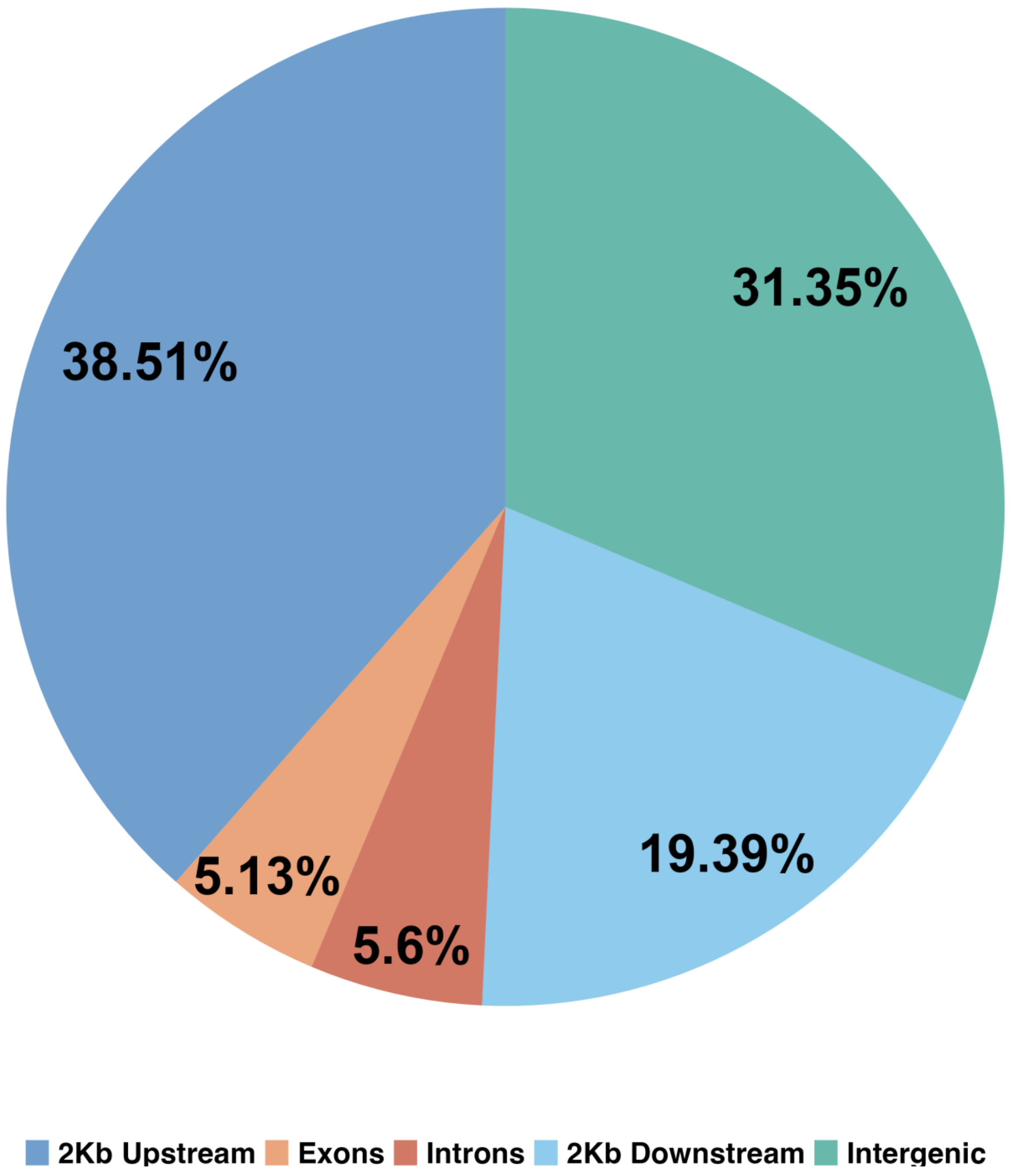

Supplementary Figure 16. ACR distribution among the genomic regions in *A. cruentus*. ACRs were called from eight *A. hypochondriacus* samples of the reference accession PI 558499 aligned to the *A. cruentus* reference genome to test for reference bias. The genome was split into 8 categories, i.e., 2 kb upstream of transcription start site (TSS), Exons, Introns, 2 kb downstream of transcription termination site (TTS) and intergenic (everything not within 2 kb of TSS or TTS of a gene)

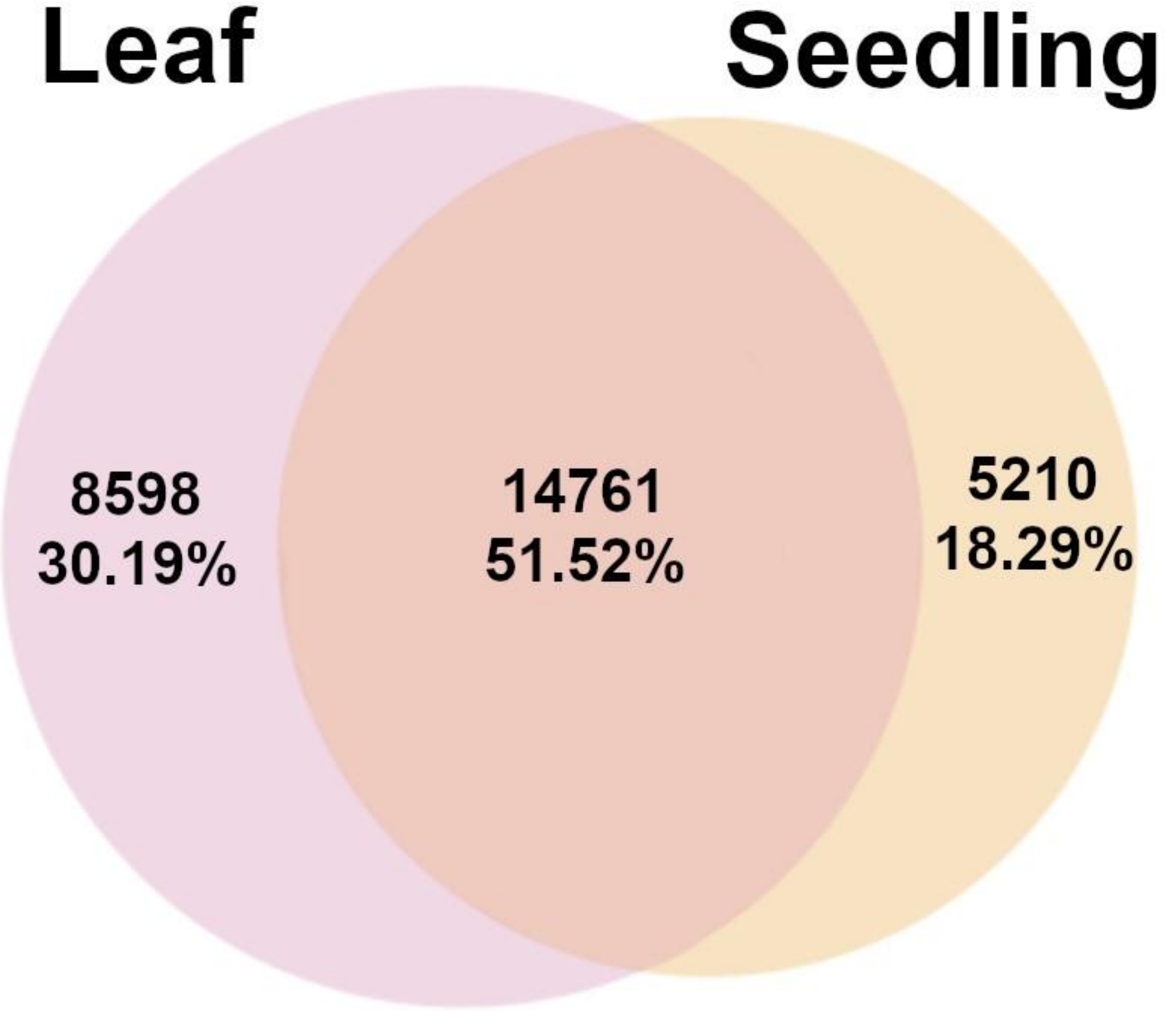

Supplementary Figure 17. Overlap of ACRs between tissues in *A. cruentus*. ACRs called from eight samples of the *A. hypochondriacus* reference accession PI 558499 aligned to the *A. cruentus* reference genome to test for reference bias. Only ACRs that occurred in at least two samples of each tissue respectively were considered. ACRs from two samples of the same tissue that overlapped were joined together. ACRs had to overlap by at least 1 bp to be considered shared between the tissues

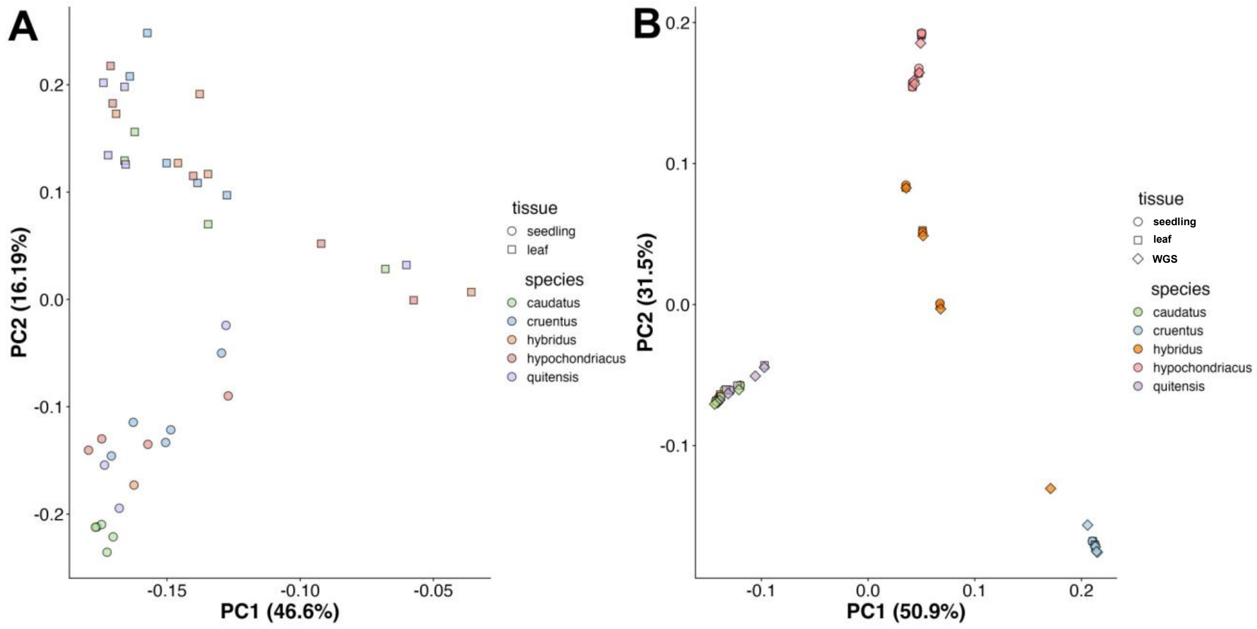

Supplementary Figure 18. Relationship between ATAC-samples based on accessible chromatin regions (ACRs) and genome-wide SNPs. PCA of the 42 ATAC-seq samples based on **A.** the 51,571 ACRs called across samples. The sampled tissue and species is indicated by shape and color, respectively. **B.** PCA based on SNP data from ATAC-sequencing data and whole genome sequencing data (WGS) showing that ATAC-seq data recovers the population structure.

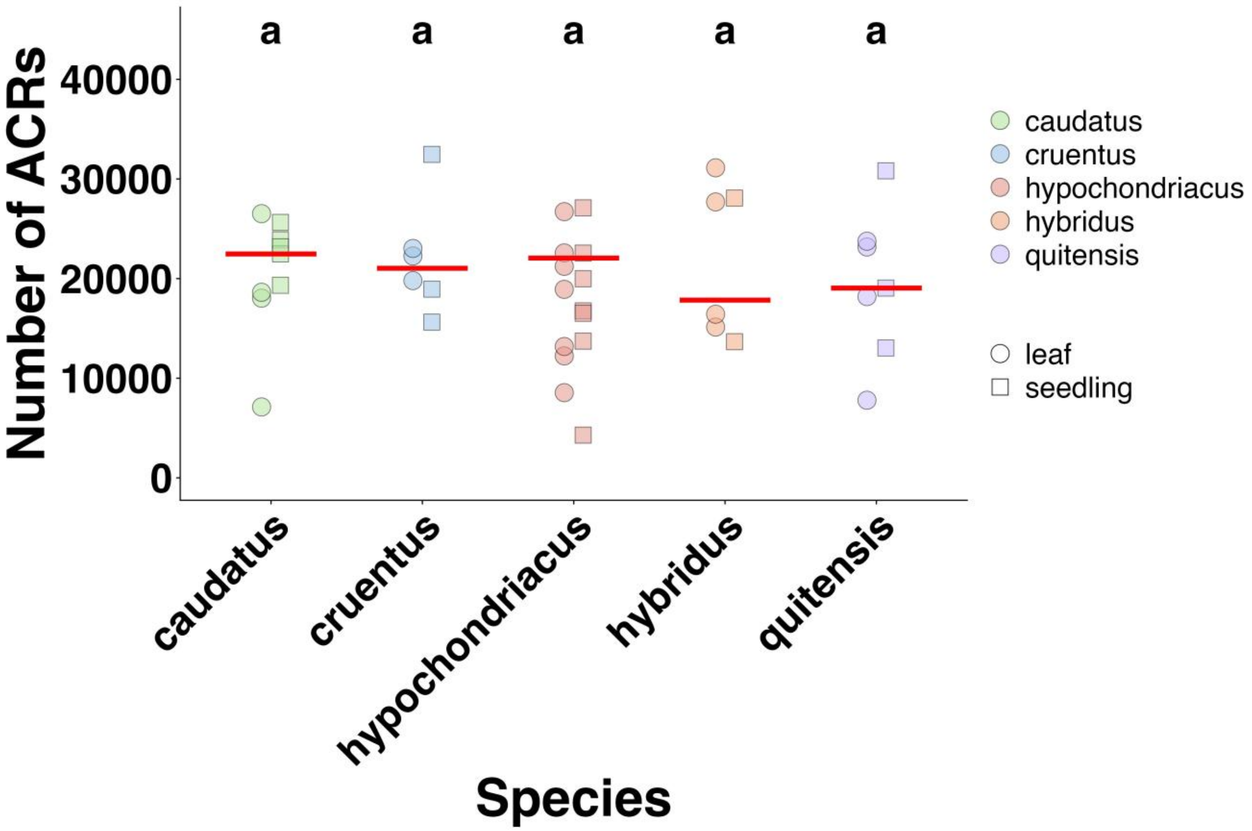

Supplementary Figure 19. Number of ACRs called for each sample when aligned to the *A. hypochondriacus* reference genome. The tissue and species from which each sample originates is indicated by shape and color, respectively. The mean is indicated by a red line for each species, respectively (*A. caudatus* n=9, *A. cruentus* n=6, *A. hypochondriacus* n= 14, *A. hybridus* n=6, *A. quitensis* n=7). Letters above the species indicate significant differences between groups based on a one-way ANOVA (species=0.535) followed by Tukey's test.

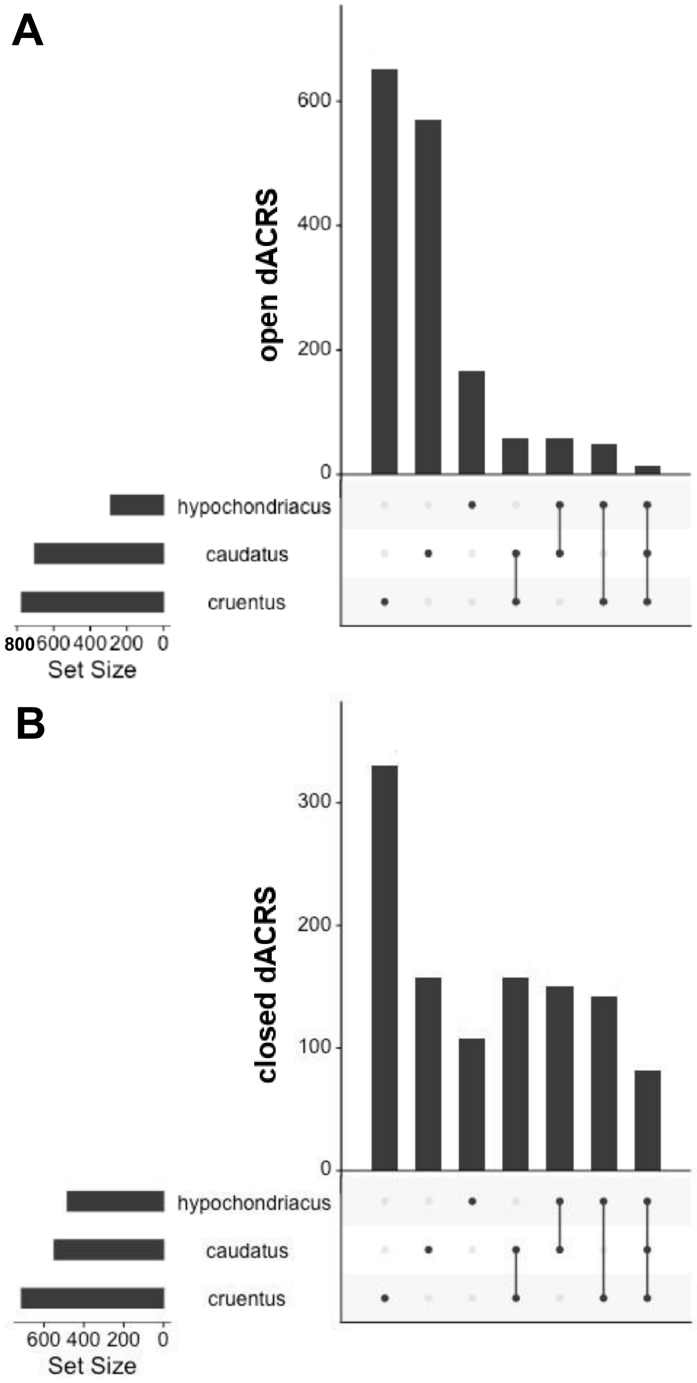

Supplementary Figure 20. Number of dACRs that were either unique to each domesticate or shared between them. UpsetR plot of ACRs that are differentially accessible (dACRs) between the domesticates and their wild ancestor. **A.** dACRs that opened during domestication. **B.** dACRs that closed during domestication.

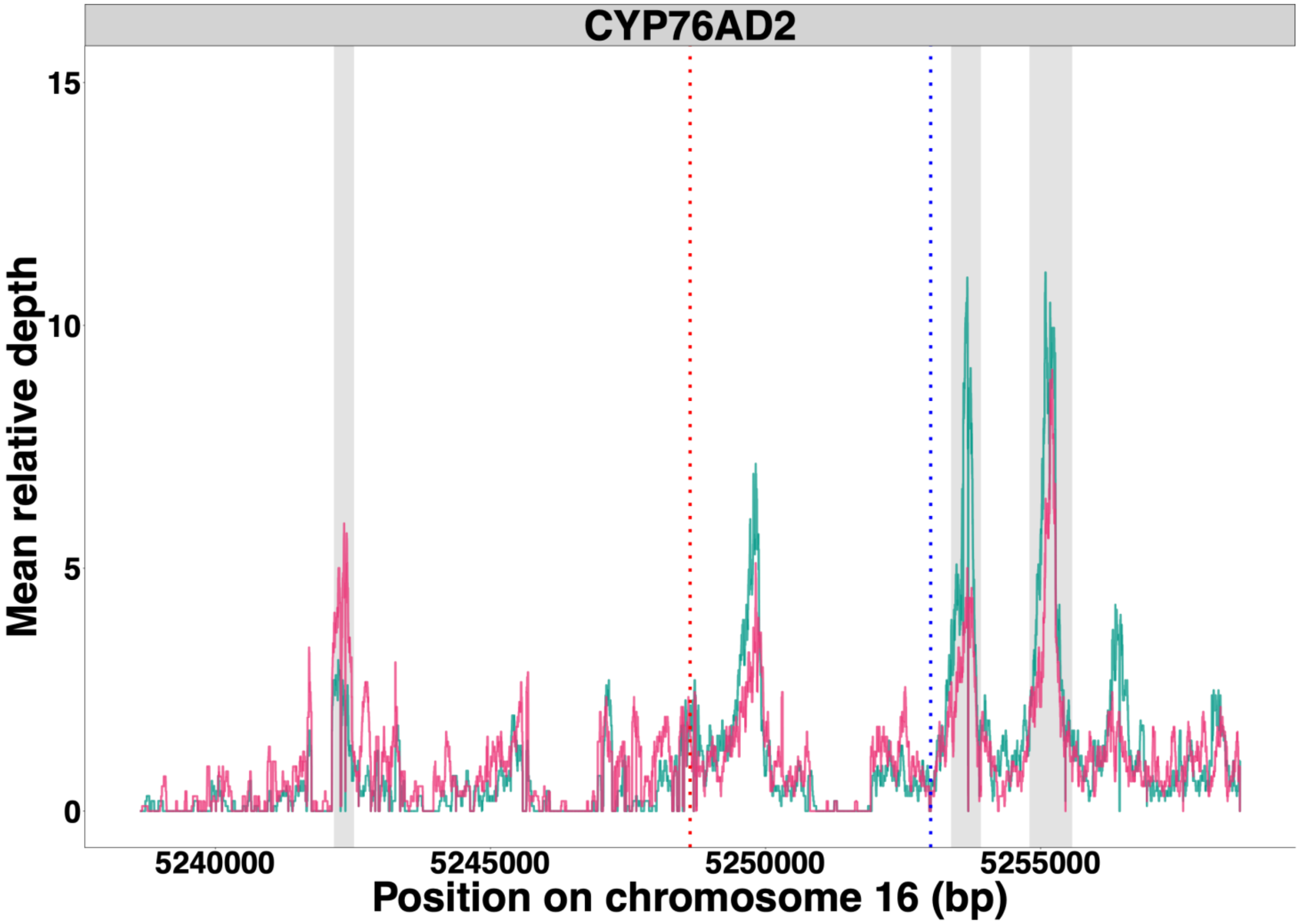

Supplementary Figure 21. Mean normalized read depth 10 kb up and downstream of the transcription start site (TSS) of CYP76AD2 for *A. caudatus* accessions with red and green seedling color (magenta and green respectively). Vertical dotted lines indicate TSS (red) and Transcription End Site (TES) (blue). Grey vertical areas indicated called accessible chromatin regions (ACRs). Normalized depth was calculated by determining the mean depth for each site across accessions with red and green seedlings respectively and normalized by the mean depth of the whole region.

Supplementary Table 1. Comparison of genome annotation statistics between published grain amaranth reference genomes.

	A. cruentus	*A. hypochondriacus* v2.2	*A. hypochondriacus* v3
Protein coding genes	25,477	23,817	25,167
Total isoforms	25,477	28,074	30,529
BUSCO score	89.8%	98.0%	98.8%
Mean CDS length (bp)	1,141	1,258	1,233
Mean exons per gene	4.86	5.39	5,21
Mean exon length (bp)	235	233	317

Supplementary Table 2. Transposable element annotation summary statistics for the *A. hypochondriacus* genome annotation v3.0. Displayed are the repetitive element class (if applicable) and subclass, the number of elements identified and the fraction of the genome they make up.

Repeat class	Element	Count	Genome (bp)	Genome (%)
LINE	L1	8,124	5,264,065	1.21
LINE	RTE	23,240	7,073,658	1.63
LTR	Copia	28,028	22,320,587	5.13
LTR	Gypsy	49,929	26,475,431	6.09
LTR	unknown	87,328	48,307,902	11.11
SINE	tRNA	44	12,510	0.00
TIR	CACTA	21,120	10,348,543	2.38
TIR	Mutator	46,252	12,834,794	2.95
TIR	PIF/Harbinger	3,888	1,626,894	0.37
TIR	TCA1/Mariner	42,218	11,476,679	2.64
TIR	hAT	50,455	16,499,746	3.79
TIR	Polinton	78	40,051	0.01
non-LTR	Penelope	62	18,384	0.00
non-TIR	Helitron	193,159	45,587,284	10.48
rDNA	45S	4,634	4,388,499	1.01
-	Repeat fragment	26,121	6,994,620	1.61
-	Total interspersed	584,680	219,269,647	50.43

Supplementary Table 3. Overview of samples used in this study.

Accession ID	Species	Sample	Mapped reads	Called peaks
PI 490518	*A. caudatus*	seedling	26324188	25478
PI 490518	*A. caudatus*	leaf	44428081	37937
PI 490518	*A. caudatus*	leaf	44672690	31659
PI 490612	*A. caudatus*	seedling	37597522	36760
PI 490612	*A. caudatus*	leaf	24522965	18941
PI 490612	*A. caudatus*	seedling	51173694	45953
PI 608019	*A. caudatus*	leaf	40953093	39437
PI 608019	*A. caudatus*	seedling	42451784	37165
PI 642741	*A. caudatus*	seedling	28311405	31401
PI 642741	*A. caudatus*	seedling	37197967	30450
PI 642741	*A. caudatus*	leaf	19980225	27455
PI 642741	*A. caudatus*	leaf	19404739	18318
PI 511714	*A. cruentus*	leaf	48802567	43012
PI 511714	*A. cruentus*	seedling	45235760	44699
PI 511717	*A. cruentus*	leaf	35981869	33253
PI 511717	*A. cruentus*	seedling	29246205	26832
PI 643058	*A. cruentus*	leaf	46230710	39692
PI 643058	*A. cruentus*	seedling	22968690	25020
PI 490489	*A. hybridus*	leaf	34286717	40907
PI 490489	*A. hybridus*	seedling	41484966	43143
PI 511754	*A. hybridus*	leaf	10576068	24806
PI 511754	*A. hybridus*	leaf	34048324	28814
PI 652426	*A. hybridus*	seedling	42521074	38959
PI 558499	*A. hypochondriacus*	seedling	32315913	22582
PI 558499	*A. hypochondriacus*	leaf	39197764	36579
PI 558499	*A. hypochondriacus*	leaf	31477634	18787
PI 558499	*A. hypochondriacus*	seedling	36857940	35456
PI 558499	*A. hypochondriacus*	leaf	27272930	29685
PI 558499	*A. hypochondriacus*	leaf	31799201	16923
PI 558499	*A. hypochondriacus*	leaf	32799196	24569
PI 558499	*A. hypochondriacus*	seedling	38503380	30749
PI 604581	*A. hypochondriacus*	leaf	34501001	31206
PI 604581	*A. hypochondriacus*	seedling	32051291	20056
PI 604587	*A. hypochondriacus*	leaf	13469060	15592
PI 604587	*A. hypochondriacus*	seedling	19992232	25289
PI 643070	*A. hypochondriacus*	seedling	30173461	30695
PI 643070	*A. hypochondriacus*	leaf	32436428	32615
PI 643070	*A. hypochondriacus*	seedling	7483949	9187
PI 490466	*A. quitensis*	seedling	46005628	39647
PI 490466	*A. quitensis*	leaf	23810641	18909
PI 511745	*A. quitensis*	leaf	42416284	36863
PI 511745	*A. quitensis*	seedling	30666794	28014
PI 652426	*A. quitensis*	leaf	40889587	39656
PI 667158	*A. quitensis*	leaf	29416173	31427
PI 667158	*A. quitensis*	seedling	25003043	25489
PI 669836	*A. quitensis*	leaf	26293386	33185

Supplementary Table 4. List of genes that were associated with dACRs with selective sweeps.

Gene ID	Gene function	Species	Domestication chromatin state
AHq003723	Belongs to the disease resistance NB-LRR family	cau	open
AHq003925	Protein NRT1 PTR FAMILY	cau	open
AHq006858	serine-type endopeptidase activity	cau	open
AHq017595	Belongs to the peroxidase family. Classical plant (class III) peroxidase subfamily	cau	open
AHq021685	Domain of unknown function (DUF4220)	cau	open
AHq021796	Reversible hydration of carbon dioxide	cau	open
AHq001071	Belongs to the 'GDSL' lipolytic enzyme family	cau	open
AHq003286	NA	cru	open
AHq010508	NA	cru	open
AHq010508	NA	cru	open
AHq014726	NA	cru	open
AHq023736	F-Box protein	cru	open
AHq001064	ammonium transporter	hyp	open
AHq003820	Protein timeless homolog	hyp	open
AHq015137	NA	hyp	open
AHq015207	caffeoyl-CoA O-methyltransferase	hyp	open
AHq024811	NA	hyp	open
AHq024812	atrbl14,rbl14	hyp	open
AHq024812	L-threonine ammonia-lyase activity	hyp	open
AHq003623	NADH dehydrogenase ubiquinone 1 beta subcomplex subunit	cau	closed
AHq006315	isoform X1	cau	closed
AHq015012	Disease resistance protein	cau, cru, hyp	closed
AHq020586	HAUS augmin-like complex subunit	cau, cru, hyp	closed
AHq020587	HAUS augmin-like complex subunit	cau, cru, hyp	closed
AHq023997	E3 ubiquitin-protein ligase SGR9	cau	closed
AHq024686	NA	cau	closed
AHq024807	NA	cau	closed
AHq024808	NA	cau, hyp	closed
AHq006411	LRR-repeat protein	cru	closed
AHq012177	NA	cru	closed
AHq015015	Disease resistance protein	cru, hyp	closed
AHq016153	NA	cru, hyp	closed
AHq016154	NA	cru, hyp	closed
AHq023818	2-dehydro-3-deoxyphosphooctonate aldolase	cru	closed
AHq023882	transcription factor	cru	closed

Supplementary references

1. Chen, H. *et al.* wgd v2: a suite of tools to uncover and date ancient polyploidy and whole-genome duplication. Bioinformatics 40, btae272 (2024).
2. Emms *et al.* Orthofinder: phylogenetic orthology inference for comparative genomics. *Genome Biology* **20**, 238 (2019).
3. Lu, Z. *et al.* The prevalence, evolution and chromatin signatures of plant regulatory elements. *Nature Plants* **5**, 1250–1259 (2019).
4. Schwope, R. *et al.* Open chromatin in grapevine marks candidate CREs and with other chromatin features correlates with gene expression. *Plant Journal* **107**, 1631–1647 (2021).
5. Clouse *et al.* The amaranth genome: genome, transcriptome, and physical map assembly. *Plant Genome* **9**, plantgenome2015–07 (2016).
